# Left Heart Dysfunction in Acromegaly Revealed by Novel Echocardiographic Methods

**DOI:** 10.3389/fendo.2020.00418

**Published:** 2020-06-24

**Authors:** Beata Uziȩbło-Życzkowska, Agnieszka Jurek, Przemysław Witek, Grzegorz Zieliński, Grzegorz Gielerak, Paweł Krzesiński

**Affiliations:** ^1^Department of Cardiology and Internal Diseases, Military Institute of Medicine, Warsaw, Poland; ^2^Department of Gastroenterology, Endocrinology and Internal Diseases, Military Institute of Medicine, Warsaw, Poland; ^3^Department of Internal Medicine, Endocrinology and Diabetes, Medical University of Warsaw, Warsaw, Poland; ^4^Department of Neurosurgery, Military Institute of Medicine, Warsaw, Poland

**Keywords:** acromegaly, left atrial strain, left ventricular strain, pituitary disease, speckle tracking echocardiography

## Abstract

**Background:** Acromegaly is a rare disease that requires modern treatment to decrease the risk of mortality, mainly from vascular diseases. Identifying acromegalic patients with increased cardiovascular risk is challenging. Speckle-tracking echocardiography (STE) is a modern, well-validated, and reproducible method of assessing left ventricular longitudinal deformation and providing a sensitive assessment of myocardial contractility. We hypothesized that STE may be useful in evaluating subclinical dysfunction of the left heart in acromegalic patients, especially when a left ventricle (LV) assessment is completed with STE of the left atrium (LA).

**Purpose:** To assess the diagnostic value of speckle-tracking echocardiography in identifying the occurrence of LV and LA functional impairment in patients with acromegaly, in comparison to patients without this rare pituitary disease.

**Methods:** Echocardiographic assessments of LV and LA function using the new STE method were performed in 60 subjects: 30 acromegalic (ACRO group) patients and a CONTROL group with 30 patients matched in terms of age, gender, systolic/diastolic pressure, and history of hypertension for at least 12 months.

**Results:** The ACRO group, compared to the CONTROL group, presented: (1) higher left ventricular mass (left ventricular mass index: 132 vs. 108 g/m^2^, *p* < 0.001) and, in consequence, more frequent LV hypertrophy (80.0 vs. 53.3%; *p* = 0.028); (2) impaired LV systolic function measured by both left ventricular ejection fraction (LVEF) (63.4 vs. 66.9%, *p* < 0.001) and global longitudinal strain (GLS) (−18.1 vs. −19.4%, *p* = 0.023); (3) greater LA anteroposterior diameter (40.3 vs. 36.9 mm, *p* = 0.003) and indexed left atrial volume (37.9 vs. 27.6 ml/m^2^, *p* < 0.001); and (4) impaired echocardiographic strain parameters corresponding with LA function.

**Conclusions:** Acromegaly, even in young patients with good blood pressure control, may be associated with left ventricular hypertrophy and subclinical impairment of the left ventricular and left atrial mechanical function, which may be identified by speckle-tracking echocardiography. Further research in this area is necessary to clarify the prognostic value of these phenomena.

## Introduction

Acromegaly is a chronic disease caused by growth factor (GH) hypersecretion. This rare pituitary disorder has some characteristic external appearance features. Unfortunately, they develop slowly and gradually, which is the cause of the relatively late diagnosis, usually after about 5–10 years and typically when the patient is around 40 years of age (1). For many years, acromegaly was associated with an increased risk of mortality, mainly from vascular diseases, due to the lack of effective treatment (2). Nowadays, the reported mortality rate in acromegaly is lower in comparison with studies published before 2008 (3). New effective treatment for acromegaly, which has developed in the last decade, has significantly improved the survival of acromegalic patients (3, 4). However, cardiovascular diseases are still one of the most frequent comorbidities in acromegalic patients, and they require early diagnosis and appropriate treatment or implementation of preventive measures (4). Prolonged tissue exposure to GH leads to significant cardiovascular remodeling. An increased level of serum GH activates cardiac growth, resulting in a hypertrophic response and, as a consequence, left ventricular (LV) diastolic, and systolic dysfunction (5, 6). It is estimated that the presence of any type of cardiovascular disease at the time of acromegaly diagnosis may triple the odds of hospitalization and may account for as many as 60–100% of deaths within the next 15 years (7). The diagnosis of symptomatic cardiovascular diseases in acromegalic patients is not difficult, whereas identifying patients with increased cardiovascular risk is more challenging.

It is worth remarking that although standard echocardiography used to be the traditionally applied technique to evaluate diastolic and systolic LV dysfunction, more recent studies encourage the use of new methods such as speckle-tracking echocardiography (STE). This modern, well-validated, and reproducible technique to assess LV longitudinal deformation offers a more sensitive assessment of myocardial contractility. Evaluating global longitudinal strain (GLS) may help to identify patients with subclinical LV systolic dysfunction. More and more new studies have revealed the importance of this method as a diagnostic tool allowing assessment of the prognosis in many cardiological and non-cardiological diseases (8–10). Our recent study showed that it is also applicable in Cushing's disease (CD) (11).

We hypothesize that STE may be useful in evaluating subclinical dysfunction of the left heart in acromegalic patients, especially when LV assessment is completed with STE of the left atrium (LA). This latter concept has arisen from reports that proved the role of STE in detecting LA dysfunction in several cardiovascular diseases (12–14). For this reason, the aim of our study was to assess the diagnostic value of STE in identifying the occurrence of LV and LA functional impairment in patients with acromegaly, in comparison to patients without this rare pituitary disease.

## Materials and Methods

### Study Design and Patients

We assessed 33 patients with acromegaly qualified for causal treatment at the Military Institute of Medicine, Poland, between 2016 and 2019 (ACRO group), diagnosed on the basis of standard hormonal criteria: an increased level of GH, increased insulin-like growth factor 1 (IGF-1), failure to suppress serum GH levels to <0.4 mcg/L during oral glucose tolerance test (OGTT), and positive pituitary MRI findings. One patient from the ACRO group was excluded from the final analysis due to the diagnosis of a significant type 2 atrial septal defect, and two patients were excluded due to non-acceptable ultrasound image quality, excluding the possibility of measuring the strain value.

The comparative control group consisted of 30 patients matched in terms of age, gender, systolic/diastolic pressure, and history of hypertension for at least 12 months.

The exclusion criteria for both groups included coronary artery disease, LVEF <50%, stroke/transient ischemic attack in anamnesis, pulmonary embolism in anamnesis, chronic obstructive pulmonary disease in severe stadium, respiratory failure, a condition after a head injury, and pregnancy.

### Standard Echocardiography

All patients underwent two-dimensional echocardiography using a high-quality echocardiograph (Vivid 7 or E95, GE, USA). Echocardiography was performed using all standard views. All measurements of LV and LA dimensions were made in accordance with the current guidelines of the European Society of Echocardiography (15). The standard parameters that were measured to assess LA function were LA end-diastolic diameter measured in the parasternal long axis (PLAX), LA area measured in the 4-chamber axis, and LA volume (LAV) and LA indexed volume (LAVI) assessed using the biplane disk summation technique from apical 4-chamber and 2-chamber views. To assess LV function, we measured LV end-diastolic diameter measured in the PLAX and LV ejection fraction (LVEF) calculated using the biplane Simpson formula in the apical 2- and 4-chamber views. LV mass (LVM) was calculated using the linear method as recommended by the American Society of Echocardiography for cardiac chamber quantification by echocardiography in adults (15). The thickness of the interventricular septal and inferolateral walls as well as LV end-diastolic and end-systolic diameters were obtained from the PLAX. To determine LV hypertrophy, LVM was indexed to body surface area (BSA) calculated using the DuBois formula.

To assess LV diastolic function, we measured waves E and A of the mitral inflow velocity, recorded using pulsed wave Doppler from the apical 4-chamber view, the ratio between peak early (E) and late (A) diastolic LV filling velocities, the velocity waves (e′ and a′) of the mitral annulus septal, and the lateral regions recorded using tissue Doppler imaging (TDI). When calculating E/e′ ratio, the average value of the septal and lateral mitral annulus velocities was used. Left ventricular diastolic dysfunction (LVDd) was diagnosed according to the current guidelines (16).

### Speckle-Tracking Echocardiography

Global and regional longitudinal two-dimensional (2D) LV and LA strain was analyzed with the speckle-tracking technique using GE EchoPAC software.

Left ventricular global longitudinal strain (GLS) was assessed with the use of automated function imaging software. Two basal points were selected at the level of the mitral annulus and the third point at the apex detection of the tracked area to carry out the assessment semi-automatically with the possibility of manual adjustments. The LV walls were divided into six segments in each apical view, and tracking quality and strain value were assessed for each LV segment. The mean global longitudinal peak systolic strain was calculated for each view. The mean of these values was the value of GLS.

For the analysis of LA strain, apical 4- and 2-chamber view images were obtained using conventional 2D gray scale echocardiography during breath hold. The frame rate was set between 60 and 80 frames per second. This technique is recommended by the Expert Consensus Statement (17). The recordings were processed using acoustic-tracking software (EchoPAC, GE, USA), allowing off-line semi-automated analysis of the speckle-based strain. The LA endocardial border was traced manually by a point-and-click method in both 4- and 2-chamber views. An epicardial border was then automatically generated by the software, creating a region of interest (ROI). After manual adjustment of the ROI shape, the software divided the ROI into six segments and generated the longitudinal strain curve. As a reference point, we set the QRS-wave onset (18) and measured the first positive peak atrial longitudinal strain (PALS), corresponding to the atrial reservoir function. We also measured strain value during early diastole, corresponding to the atrial conduit function [in this study, called conduit atrial longitudinal strain (CALS)] and the second positive peak atrial longitudinal strain (strain during late diastole), corresponding to active atrial contraction and called peak atrial contraction strain (PACS). PALS and PACS were calculated by averaging the values observed in all LA segments (global PALS and global PACS) and by separately averaging the values observed in the 4- and 2-chamber views (PALS/PACS apical 4-chamber—PALS/PACS A4C and PALS/PACS apical 2-chamber—PALS/PACS A2C). CLS was calculated by averaging the values observed in all LA segments (global CLS). The time to peak longitudinal strain (TPLS) was measured as the average of all 12 segments (global TPLS) and by separately averaging the values observed in the two apical views (TPLS apical 4-chamber—TPLS A4C and TPLS apical 2-chamber—TPLS A2C). When some segments were excluded due to the inability to achieve adequate tracking, PALS and TPLS were calculated by averaging the values measured in the remaining segments.

**Figure 1:**
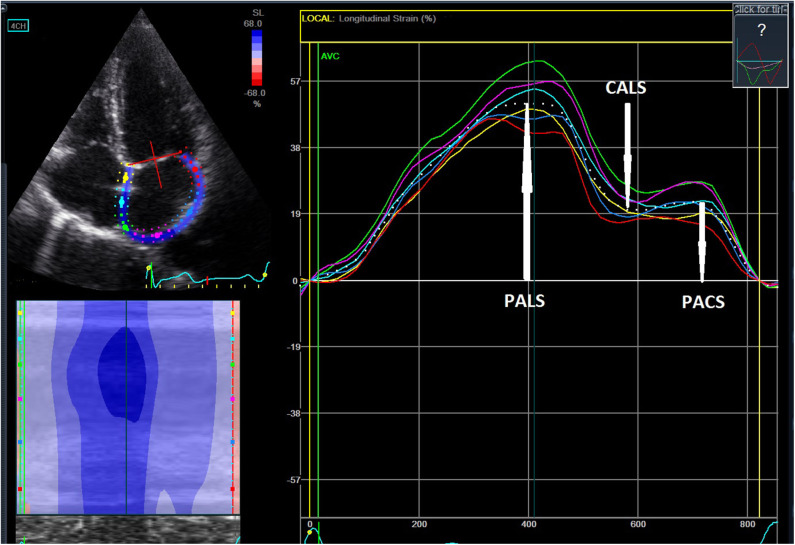
Assessment of left atrial strain referenced to the QRS-wave onset of the electrocardiogram. The peak positive longitudinal strain corresponds to the atrial reservoir function (peak atrial longitudinal strain—PALS), the strain during early diastole corresponds to the atrial conduit function (conduit longitudinal strain—CALS), and the strain during late diastole reflects the atrial booster pump function (peak atrial contractile strain—PACS).

Figure 1 presents the method for measuring PALS, PACS, and CALS.

The intraobserver variability of PALS 4CH, PALS 2CH, PACS 4CH, and PACS 2CH was assessed in 20 randomly selected patients. Intraobserver variability coefficients were calculated using images independently recorded on two different occasions by the same investigator. The intraclass correlation coefficient together with the mean difference (95% CI) of two measurements in Bland–Altman analysis, divided by the mean of those two measurements and given as percentages, was calculated for intraobserver variability.

### Statistical Analysis

Statistical analysis was performed using Statistica 12.0 (StatSoft, Inc., Tulsa, U.S.). The distribution and normality of the data were assessed using the Kolmogorov–Smirnov test. The categorical variables were presented as absolute and relative frequencies (percentages), and continuous variables were presented as means ± standard deviation (SD). The ACRO and CONTROL groups were compared in terms of clinical, echocardiographic, and hemodynamic parameters with the use of the Student's *t*-test/Mann–Whitney *U*-test for continuous variables and chi-squared or Fisher's exact test for categorical variables. A *p-*value of <0.05 was considered statistically significant.

## Results

### Clinical Characteristics

Thirty patients in the ACRO group [17 males (56.6%); mean age 47 ± 13 years; age range 20–66 years] had acceptable ultrasound image quality and were included in the final analysis. The CONTROL group comprised 30 patients [14 males (46.6%); mean age 48 ± 9 years; age range 26–65 years]. There were no significant differences between the groups in terms of gender, age, concomitant diseases, or pharmacotherapy for arterial hypertension (AH), which was the only cardiovascular disease in most patients. Acromegalic patients had a higher resting heart rate (HR) compared to the CONTROL group (76.4 vs. 65.9/min; 0 = 0.0001). In both groups, blood pressure was well controlled (ACRO: 122/78 mmHg; CONTROL: 121/77 mmHg). Five patients in the ACRO group were diagnosed with diabetes mellitus (DM) type 2, which was well controlled. Moreover, the duration of DM 2 was less than a year, and patients were treated either with a low dose of only one antidiabetic drug—metformin (three patients)—or diet alone (two patients). The duration of DM 2 was determined on the basis of medical history or laboratory test. For three patients, we had the fasting glucose result obtained 9–12 month earlier, and for the other two patients, the history of antidiabetic treatment was <12 months. None of the patients in either group had chronic kidney disease, coronary artery disease, atrial fibrillation (AF), stroke in anamnesis, or pulmonary diseases. The detailed baseline clinical characteristics of both groups and the average GH and IGF-1 levels measured in the acromegalic patients are presented in Table 1.

**Table 1:** Baseline clinical characteristics for the two study groups.

	**CONTROL group**	**ACRO group**	***P*-value**
Age; mean(SD)	47.9 (9.6)	46.9 (13,3)	0.73
Male; *n* (%)	14 (46.7)	16 (53.3)	0.61
BMI; mean (SD)	28.6 (4.3)	27.9 (4.1)	0.52
HR; mean (SD)	65.9 (9.3)	76.4 (10.5)	<0.001
SBP; mean (SD)	121.9 (9.6)	120.8 (11.7)	0.69
DBP; mean (SD)	78.4 (8.7)	77.2 (9.7)	0.57
Creatinine; mean (SD)	0.85 (0.2)	0.75 (0.2)	0.04
AH; *n* (%)	20 (66.7)	17 (56.7)	0.42
**Antihypertensive treatment**, ***n*** **(%)**			
ACEI	12 (40.0)	12 (40.0)	1.00
ARB	4 (13.3)	1 (3.3)	0.16
Diuretics	7 (23.3)	4 (13.3)	0.32
BB	2 (6.7)	6 (20.0)	0.13
CCB	5 (16.7)	8 (26.7)	0.35
**Hormone measurements (basic conditions)**			
GH (ng/mL); median	–	12.7	–
IGF-1 (ng/mL); median	–	472.3	–
IGF-1 (ULN); mean (SD)	–	2.28 (1.23)	–

*ACEI, angiotensin converting enzyme inhibitors; AH, arterial hypertension; ARB, angiotensin receptors blocker; BMI, body mass index; BB, beta blockers; CCB, calcium channel blockers; DBP, diastolic blood pressure; GH, growth hormone; IGF-1, insulin-like growth factor; HR, heart rate; SBP, systolic blood pressure*.

### Echocardiographic Evaluation of Left Ventricular Function

Compared to the CONTROL group, the ACRO group presented higher left ventricular mass (LVMI: 132 vs. 108 g/m^2^, *p* < 0.001) and, in consequence, more frequent LV hypertrophy (80.0 vs. 53.3%; *p* = 0.028). The occurrence of LVDd, measured according to the standard parameters, tended to be higher in the ACRO group, but the difference reached only borderline statistical significance (13.3 vs. 33.3%, *p* = 0.067). Patients in the ACRO group had lower e′ septal value (8 vs. 9.9 cm/s; *p* = 0.004), but no differences were noted for e′ lateral value (10.9 vs. 12.3 cm/s; *p* = 0.08) and E/e′ ratio (6.3 vs. 6.9; *p* = 0.28). Acromegaly was also related to impaired LV systolic function. There were differences between the ACRO and the CONTROL group both in the assessment of LVEF (63.4 vs. 66.9%, *p* < 0.001) and GLS (mean: −18.1 vs. −19.4%, *p* = 0.023; median [interquartile range (IQR)]: 18.5% (13.0–22.9%) vs. 19.8 (14.5–25.3, *p* = 0.02) (see Table 2). Even after excluding the five diabetic patients from the ACRO group, these differences remained significant (see Table S1).

**Table 2:** Echocardiographic parameters for the two study groups.

	**CONTROL group**	**ACRO group**	***p***
**Basic echocardiography parameters**			
RVEDd (mm); mean (SD)	29.9 (4.01)	31.8 (3.88)	0.07
IVSDd (mm); mean (SD)	9.6 (1.43)	10.6 (1.88)	0.11
LVEDd; mean (SD)	48.9 (4.44)	51.9 (4.88)	0.01
PWDd (mm); mean (SD)	9.9 (1.81)	10.8 (1.69)	0.02
Aorta asc. (mm); mean (SD)	31.2 (3.30)	32.6 (4.22)	0.14
RWT; mean (SD)	0.4 (0.05)	0.4 (0.07)	0.38
RWT > 0.45; *n* (%)	3 (10.0)	8 (26.7)	0.09
**LV hypertrophy**			
LVM; mean (SD)	174.3 (54.6)	215.9 (64.40)	0.009
LVMI (g/m^2^); mean (SD)	108.6 (24.3)	132.5 (28.37)	<0.001
LVH; *n*(%)	16 (53.3)	24 (80.0)	0.03
**Echocardiography parameters of LV diastolic function**			
E/A; mean (SD)	1.15 (0.4)	1.03 (0.4)	0.14
DcT E; mean (SD)	194.5 (45.8)	213.6 (53.9)	0.07
E′m; mean (SD)	9.9 (2.9)	8.0 (2.7)	0.004
E′l; mean (SD)	12.3 (3.5)	10.9 (2.6)	0.08
E; mean (SD)	72.1 (13.9)	56.9 (12.7)	<0.001
E/E′; mean (SD)	6.9 (2.1)	6.3 (1.7)	0.28
E/A <0.8; *n*(%)	4 (13.3)	12 (40.0)	0.02
E′m <7; *n*(%)	5 (16.7)	8 (26.7)	0.35
E′l <10; *n*(%)	6 (20.0)	10 (33.3)	0.24
LVDd; *n*(%)	4 (13.3)	10 (33.3)	0.07
**Echocardiography parameters of LV systolic function**			
GLS (%); mean (SD)	19.4 (2.4)	18.1 (2.02)	0.02
LVEF (%); mean (SD)	66.9 (2.8)	63.4 (3.8)	<0.001
S′m; mean (SD)	8.8 (1.66)	7.9 (1.4)	0.03
S′l; mean (SD)	10.5 (2.03)	9.9 (2.4)	0.22

*Aorta asc, aorta ascendens diameter; GLS, global longitudinal strain; IVSDd, intraventricular septum diastolic diameter; PWDd, posterior wall end-diastolic diameter; LV, left ventricle; LVDd, left ventricular diastolic dysfunction; LVEDd, left ventricular end-diastolic diameter; LVEF, left ventricular ejection fraction; LVH, left ventricular hypertrophy; LVM, left ventricular mass; LVMI, left ventricular mass index; RVEDd, right ventricular end-diastolic diameter; RWT, relative wall thickness*.

### Echocardiographic Evaluation of Left Atrial Function

The echocardiographic parameters assessing LA morphology in the ACRO group, compared to the CONTROL group, were different. The ACRO subjects presented a greater LA anteroposterior diameter (40.3 vs. 36.9 mm, *p* = 0.003) and indexed LAV (LAVI) (37.9 vs. 27.6 ml/m^2^, *p* < 0.001; see Table 3).

**Table 3:** Data for the echocardiographic left atrial function parameters, including strain values for patients with and without diagnosed acromegaly.

	**CONTROL group**	**ACRO group**	***p***
**Standard echocardiography parameters**			
LAd (mm); mean (SD)	36.8 (4.39)	40.6 (4.4)	<0.001
LA enlargement (from LAd); *n* (%)	7 (23.3)	18 (60.0)	0.004
LAV (ml); mean (SD)	53.1 (15.04)	75.3 (15.69)	<0.001
LAVI (ml/m^2^); *n* (SD)	27.5 (6.1)	37.9 (6.6)	<0.001
LA enlargement (from LAVI); *n* (%)	3 (10.0)	23 (76.7)	<0.001
**Speckle tracking echocardiography 2D parameters**			
PALS GL (%); mean (SD)	36.2 (6.6)	26.2 (6.1)	<0.001
PALS A4C (%); mean (SD)	35.01 (7.8)	24.9 (6.9)	<0.001
PALS A2C (%); mean (SD)	37.4 (7.8)	27.4 (6.6)	<0.001
PACS GL (%); mean (SD)	17.8 (4.3)	12.8 (3.7)	<0.001
PACS A4C (%); mean (SD)	16.9 (5.3)	11.8 (3.4)	<0.001
PACS A2C (%); mean (SD)	18.7 (4.9)	13.8 (4.7)	<0.001
TPLS GL (ms); mean (SD)	399.2 (30. 1)	431.9 (39.9)	<0.001
TPLS A4C (ms); mean (SD)	408.8 (33.2)	445.7 (45.3)	<0.001
TPLS A2C (ms); mean (SD)	389.7 (32.9)	418.1 (41.5)	<0.001
CALS GL (%); mean (SD)	18.4 (5.1)	13.4 (4.9)	<0.001

*A2C, apical 2-chamber view; A4C, apical 4-chamber view; CALS, conduit longitudinal strain; GL, global; LA, left atrial; LAD, left atrium diameter; LAV, left atrium volume; LAVI, left atrium volume index; PACS, peak atrial contraction strain; PALS, peak atrial longitudinal strain; TPLS, time to peak longitudinal strain*.

All echocardiographic strain parameters corresponding with LA function were impaired in the ACRO group. Patients with diagnosed acromegaly presented significantly lower LA global PALS, PACS, and CALS, as well as separately averaged values observed in the 4- and 2-chamber views (PALS/PACS A4C and PALS/PACS A2C). Table 3 details the pooled data for the echocardiographic LA parameters, including the strain values of patients with and without diagnosed acromegaly. Even after excluding the five diabetic patients from the ACRO group, these differences remained significant (see Table S1).

The repeatability of the LA strain measurements was very high. The intraclass correlation coefficient for intraobserver variability of LA strains was as follows: for PALS A4C−0.99, PALS A2C−0.98, PACS A4C−0.99, and PACS A2C−0.98. The mean difference divided by the mean of two measurements for intraobserver variability was as follows: for PALS A4C−0.4% (−1.1–2.0%), PALS A2C−0.6% (−1.6–2.8%), PACS A4C−0.2% (−3.7–6.6%), PACS A2C−0.5% (−3.1–4.1%).

## Discussion

Our study revealed that STE may be useful in evaluating subclinical dysfunction of the left heart in acromegalic patients. We confirmed the clinical value of STE in LV assessment and revealed even more expressed impairment of LA deformation. To our knowledge, this is the first such report. The comparison of relatively young acromegalic patients, seemingly without tangible cardiological burden, to controls without acromegaly enabled the identification of strongly expressed heart abnormalities.

### Left Ventricle Morphology and Function

An elevated level of GH in acromegaly stimulates the liver to synthesize increased levels of IGF-1. Both GH and IGF-1 may cause acromegalic morphological and functional changes, either directly by affecting myocyte growth and contractility or indirectly by affecting peripheral vascular resistance, which modifies extracellular volume and neurohormonal activity (19, 20). Both of these pose a risk of LV diastolic and systolic dysfunction. Recent studies have shown impaired LV systolic function in acromegalic patients assessed using both standard methods and new echocardiography methods such as speckle-tracking echocardiography (21, 22). In our study, the ACRO group presented a higher LV mass, impaired LV systolic function, and mitral annulus velocity (e′). We found LV myocardial hypertrophy in 80% of our acromegalic subjects, but only 56.7% of them were diagnosed with AH. This proves that acromegaly itself leads to LV hypertrophy, even though blood pressure is not elevated.

We also revealed that acromegalic patients have significantly impaired LV contractility, presented especially by significantly lower GLS value. In our previous study in patients with another pituitary disease (Cushing's disease), we revealed that they present more pronounced LV systolic and diastolic dysfunction than hypertensive patients and healthy individuals (11). Patients with CD had significantly lower LV contractility expressed by GLS, despite comparable LVEF. These observations suggest cardiovascular complications due to cortisol excess that may be partly similar to those depending on GH/IGF-1. It is necessary to clearly specify that although the differences between LVEF in both groups were statistically significant, its values were in the normal range for both groups. However, significantly lower GLS in the acromegalic patients is worth mentioning. The reports on GLS in acromegaly are not entirely consistent. Popielarz-Grygalewicz et al. (21) examined 140 acromegalic patients with normal LV systolic function measured by LVEF with GLS slightly lower than in the control group (19.2 vs. 20.7%; *p* < 0.01). Di Bello et al. (23) found that the acromegalic heart showed impaired LV systolic function assessed by systolic strain and systolic strain rate. However, a study by Volschan et al. (24), which evaluated left ventricular longitudinal strain in 37 acromegalic patients compared to 48 controls, did not show impaired LV systolic function assessed by GLS. It is quite difficult to explain the differences in the results obtained without precise data about the stage of the disease. Maybe this is the reason for the differences; in our study, the GH median in the ACRO group was 12.7 vs. 8.64 ng/mL in Volschan et al. (24).

### Left Atrial Morphology and Function

The most important novelty of our work concerns the LA dysfunction in the ACRO group. We evaluated LA morphology and its several important functions: (1) as a booster pump during late ventricular diastole (PACS), (2) as a reservoir for the inflow volume received from the pulmonary veins during ventricular systole and isovolumic relaxation (PALS), and (3) as a passive conduit during early ventricular diastole and diastasis (CALS) (25). The ACRO patients presented impaired LA morphology, including greater LA anteroposterior diameter and LAVI than the controls. These findings are consistent with other studies. Popielarz-Grygalewicz et al. (21) also noted significant LA enlargement in acromegalic patients (LAVI 41.4 vs. 29.9 ml/m^2^; *p* < 0.001).

Even more impressive are the results of the LA strain parameters. Patients with acromegaly presented significantly lower LA global PALS, PACS, and CALS as well as separately averaged values observed in 4- and 2-chamber views (PALS/PACS A4C and PALS/PACS A2C). This indicates a significant impairment of the LA function in all its phases: as the reservoir, conduit, and booster pump. We did not find another study that assessed LA function in acromegalic patients by using 2-dimensional STE (2D-STE). Kormányos et al. (26) evaluated 19 acromegaly patients using both standard and three-dimensional STE (3D-STE) and found that LA volumes and LA strain were significantly different between all the acromegaly patients and the controls. They detected increased peak global and mean segmental radial and 3D strains and decreased LA circumferential strain in all the acromegaly patients as compared to the healthy subjects. It is obvious, however, that we cannot directly compare the results of the 2D STE findings with the 3D ones.

LA pathologies are robust predictors of adverse cardiovascular outcomes such as stroke, congestive heart failure, and cardiovascular death (27, 28). Just the impairment of LA mechanical function and not necessarily its enlargement significantly increases the risk of arrhythmias, mainly AF (12, 29, 30). Meanwhile, it is well-known that acromegaly is associated with a higher risk of ectopic beats, paroxysmal AF, and supraventricular tachycardia (18). Therefore, we can hypothesize that patients with acromegaly and impaired LA function may be candidates for periodic electrocardiographic assessment, including long-term telemonitoring, to actively search for subclinical arrhythmia.

### Clinical Implications

Two-dimensional STE analysis allows assessment of both the LV and LA deformation profile during an entire cardiac cycle. This method, which closely follows LV and LA physiology, is also relatively quick and easy to perform (14). The usefulness of this novel method is evident in the prediction of subclinical cardiac dysfunction in many diseases, as was presented above. Early identification of left heart functional disturbances may be the key to optimizing treatment and reducing mortality in acromegaly.

### Limitations

We realize that the small sample size is a limitation of this study. However, it should be borne in mind that acromegaly is a rare disease. On the other hand, the strength of our results lies in the fact that we recruited acromegalic subjects with no other serious chronic cardiovascular diseases apart from AH. The duration of hypertension, which was not investigated in detail, might potentially confound the results. Our study mainly involved young and middle-aged patients, and our results should not be extrapolated to the general population. Another limitation of our study is the fact that five subjects with acromegaly were burdened with DM type 2. However, all of them were successfully treated with low doses of oral antidiabetic drugs. Finally, it is worth mentioning that the role of STE in the assessment of LA deformation dynamics is still not well established. This new echocardiographic method was initially developed for LV function assessment and only recently has it been applied to assess LA deformation.

## Conclusions

Acromegaly, even in young patients with good blood pressure control, may be associated with left ventricular hypertrophy and subclinical impairment of the left ventricular and left atrial mechanical function, which may be identified by speckle-tracking echocardiography. Further research in this area is necessary to clarify the prognostic value of these phenomena.

## Data Availability Statement

The datasets generated for this study are available on request to the corresponding author.

## Ethics Statement

The studies involving human participants were reviewed and approved by The Ethics Committee of the Military Institute of Medicine in Warsaw. The patients/participants provided their written informed consent to participate in this study.

## Author Contributions

BU-Ż conceived the concept of the study. BU-Ż and PK contributed to the study design. BU-Ż, AJ, PW, and GZ organized the database. PK performed the statistical analysis. BU-Ż and PK wrote the manuscript. All authors contributed to manuscript revision, read, and approved the submitted version.

## Conflict of Interest

The authors declare that the research was conducted in the absence of any commercial or financial relationships that could be construed as a potential conflict of interest. The handling Editor declared a past co-authorship with one of the authors GZ.
