# Supplementary material for: Left Heart Dysfunction in Acromegaly Revealed by Novel Echocardiographic Methods
Source: Front Endocrinol (Lausanne). 2020 Jun 24;11:418. doi: 10.3389/fendo.2020.00418 (PMC7326767; doi:10.3389/fendo.2020.00418)
Supplement: Supplementary file 1 [file Table_1.docx]

|  | CONTROL group; n=30 | | non-diabetic ACRO group; n=25 | p |
| --- | --- | --- | --- | --- |
| Demographic data | | | | |
| Age (year); mean(SD) | 47.9 (9.6) | 45.3 (13.8) | | 0.40 |
| Male; n(%) | 14 (46.7) | 13 (52) | | 0.69 |
| Hypertension, n(%) | 20 (66.7) | 12 (48) | | 0.16 |
| Standard echocardiography parameters | | | | |
| LAd (mm); mean (SD) | 36.8 (4.39) | 39.3 (3.76)) | | 0.03 |
| LAV (ml); mean (SD) | 53.1 (15.04) | 74.2 (16.4) | | <0.001 |
| LAVI (ml/m^2^); n(SD) | 27.5 (6.1) | 37.6 (7.1) | | <0.001 |
| LVMI (g/m^2^); mean (SD) | 108.6 (24.3) | 129 (26.5) | | 0.008 |
| LVEF (%); mean(SD) | 66.9 (2.8) | 63.7 (3.6) | | <0.001 |
| Speckle tracking echocardiography 2D parameters | | | | |
| PALS GL (%); mean (SD) | 36.2 (6.6) | 26.3 (5.9) | | <0.001 |
| PALS A4C (%); mean (SD) | 35.01 (7.8) | 24.8 (6.9) | | <0.001 |
| PALS A2C (%); mean (SD) | 37.4 (7.8) | 27.7 (6.4) | | <0.001 |
| PACS GL (%); mean (SD) | 17.8 (4.3) | 12.6 (3.5) | | <0.001 |
| PACS A4C (%); mean (SD) | 16.9 (5.3) | 11.6 (3.1) | | <0.001 |
| PACS A2C (%); mean (SD) | 18.7 (4.9) | 13.7 (4.7) | | <0.001 |
| TPLS GL (ms) ; mean (SD) | 399.2 (30.1) | 425.8 (37.0) | | 0.005 |
| TPLS A4C (ms) ; mean (SD) | 408.8 (33.2) | 439.3 (42.2) | | 0.004 |
| TPLS A2C (ms) ; mean (SD) | 389.7 (32.9) | 412.3 (39.9) | | 0.03 |
| CALS GL (%); mean (SD) | 18.4 (5.1) | 13.7 (5.1) | | <0.001 |
| LV GLS (%); mean(SD) | 19.4 (2.4) | 18.4 (1.7) | | 0.04 |
| Abbreviations: A2C, apical 2-chamber view; A4C, apical 4-chamber view; CALS, conduit atrial longitudinal strain; GL, global; LA, left atrial; LAD, left atrium diameter; LAV, left atrium volume; LAVI, left atrium volume index; LVEF, left ventricular ejection fraction; LV GLS, left ventricular global longitudinal strain; PACS, peak atrial contraction strain; PALS, peak atrial longitudinal strain; TPLS, time to peak longitudinal strain. | | | | |

Table S1. Comparison of the CONTROL group with the non-diabetic ACRO subjects (after excluding 5 patients with diabetes mellitus type 2 ).
